# P15 Maintaining good antimicrobial stewardship on the virtual ward

**DOI:** 10.1093/jacamr/dlaf239.019

**Published:** 2026-01-05

**Authors:** Yasmin Elmasry, Austin Trotman, Katie Heard

**Affiliations:** Pharmacy Department, University Hospitals Plymouth NHS Trust, Plymouth, UK; Pharmacy Department, University Hospitals Plymouth NHS Trust, Plymouth, UK; Pharmacy Department, University Hospitals Plymouth NHS Trust, Plymouth, UK

## Abstract

**Background:**

A tertiary hospital in South West England has implemented a virtual ward service allowing patients to complete courses of IV antibiotics at home, in lieu of a commissioned outpatient parenteral antimicrobial therapy (OPAT) service. This model facilitates admission avoidance, earlier discharge and continuity of care while maintaining patient safety. In the absence of a formal OPAT service, robust antimicrobial stewardship oversight is crucial to ensure the appropriate use of antimicrobials. As such, only patients with an antimicrobial plan approved by an infection specialist are accepted for virtual ward treatment.

**Objectives:**

Between April and September 2025, data were collected to evaluate the number and outcomes of virtual ward referrals to the AMS team for IV antimicrobial therapy. The primary aim was to assess acceptance rates and identify common reasons for rejection to inform service improvement and support prudent antimicrobial prescribing.

**Methods:**

All virtual ward referrals for IV antimicrobials during the study period were prospectively reviewed by the AMS pharmacists. Data were recorded on referral volumes, acceptance and rejection rates, reasons for rejection, antimicrobial agents requested, and clinical indications. Rejections were categorized by decision-maker (AMS pharmacist, microbiology consultant, or virtual ward team) and underlying rationale, including suitability for oral switch, patient fitness for discharge, or inappropriate referral.

**Results:**

Over the 6 month period, 162 referrals were made for IV antimicrobials. Of these, 98 (60%) were accepted and 64 (40%) declined. AMS pharmacists declined the majority of referrals (23), most commonly due to the availability of suitable oral alternatives (15), patients not being medically fit for discharge (3), antibiotics stopped (4). Microbiology declined 10 referrals, due to availability of appropriate oral options (5) and pending ongoing investigations (5). The virtual ward team declined 31 referrals, mainly due to medical unfitness for discharge (20), inappropriate referrals (4) or home environment deemed inappropriate (2). The most frequent indications among declined referrals were cellulitis (19) due to oral options being available. Bacteraemia referrals (15) were declined due to no confirmed source or pending investigations which were similar reasons as endocarditis (4), intra-abdominal abscess (6) and bacterial meningitis (4).

**Conclusions:**

This evaluation highlights the essential role of AMS and infection specialist oversight in ensuring safe and appropriate antimicrobial use within a virtual ward model. A significant 40% of referrals were declined, most often due to the availability of suitable oral therapy or patient unfitness for discharge, underscoring the need for early AMS input during inpatient care. However, complex patients remain outside the remit of virtual ward and make a case for a formalized and commissioned OPAT service which adheres to BSAC good practice recommendations.

